# Human non-decalcified histology of three dental implants 45 months under function—a case report

**DOI:** 10.1186/s40729-019-0184-4

**Published:** 2019-09-25

**Authors:** Rafael Silveira Faeda, Suzana Clesia Silverio do Nascimento, Pâmela Leticia Santos, Rodolfo Jorge Boeck, Rafael Sartori, Rogério Margonar, Elcio Marcantonio

**Affiliations:** 1 Department of Health Sciences, Post-graduation Program in Implantology, University of Araraquara - UNIARA Dental School, Rua Carlos Gomes, 1338, Centro, Araraquara, SP 14801-340 Brazil; 2 Department of Health Sciences, University of Araraquara (UNIARA), Araraquara, SP Brazil; 3 Department of Diagnosis and Surgery, School of Dentistry at Araraquara, São Paulo State University (UNESP), Araraquara, SP Brazil

**Keywords:** Dental implants, Circularly polarized light, Collagen fiber orientation, Tooth-implant connection

## Abstract

**Background:**

Fracture of an implant is a quite rare event but represents an important opportunity to evaluate the peri-implant bone tissue response to implant overload in human beings. This study aimed to evaluate bone tissue around three fractured titanium implants retrieved from a human maxilla, by histomorphometric and birefringence analyses.

**Case report:**

For this, the implants and the surrounding bone were removed after having been united to a tooth in function for 45 months, by a 4-mm internal diameter trephine bur, following an undecalcified section was obtained. The results showed a rate of 77.3% of bone-to-implant contact (BIC) and 80.3% of bone area filling within the limits of the implant threads. Under circularly polarized light microscopy investigation, the amount of the transverse collagen fibers was of 48.11%, and the amount of the longitudinal collagen fibers was of 51.89%.

**Conclusion:**

Within the limitation of this study, the possible cause of the implant fracture could be the association of overload, inadequate implant diameter, and fragile internal hexagon connection.

## Background

The osseointegration process is defined as the direct contact of living bone and a loaded implant at the microscopic level [1]. This event has been shown by different animal models [2–4] and in a few human histological reports [5], being influenced by many variable like systemic conditions (diabetes mellitus [3] and cigarette smoking [4]), implant surface macro- and microtexture (roughness and treatment methods) [6, 7], and prosthetic restoration stability [8].

The Branemark protocol recommends the isolation of the implants from the natural teeth abutments for partially edentulous situations, due to the potential difference in the way natural teeth and implants would react to static and dynamic loading [9]. While a natural tooth with a healthy periodontal ligament has a mobility of 50 to 200 μm, an osseointegrated implant may move only 10 μm, which is primarily a result of bone flexibility [10]. Eventually, there are different stress and strain patterns in the bone surrounding an implant compared with a natural tooth under masticatory forces [10]. The clinical outcomes associated with this problem include bone resorption around the implant neck, bone or implant fracture, fracture of attachment screws, loosening of attachment screws, cement failure, prosthetic cantilever comportment, and intrusion of a natural tooth [11].

Implant fracture is the most catastrophic failure of implant components because it usually causes the loss of the implant and prosthesis. However, an osseointegrated fractured implant represents a very useful opportunity to study, in human beings, the effects of overloading of the peri-implant bone microstructure [8]. The aim of this case report was to describe three sandblasted and acid-etched implants fractured after 45 months of load, and analyze the peri-implant bone microstructure.

## Clinical report

### Implant specimen

**Fig. 1:**
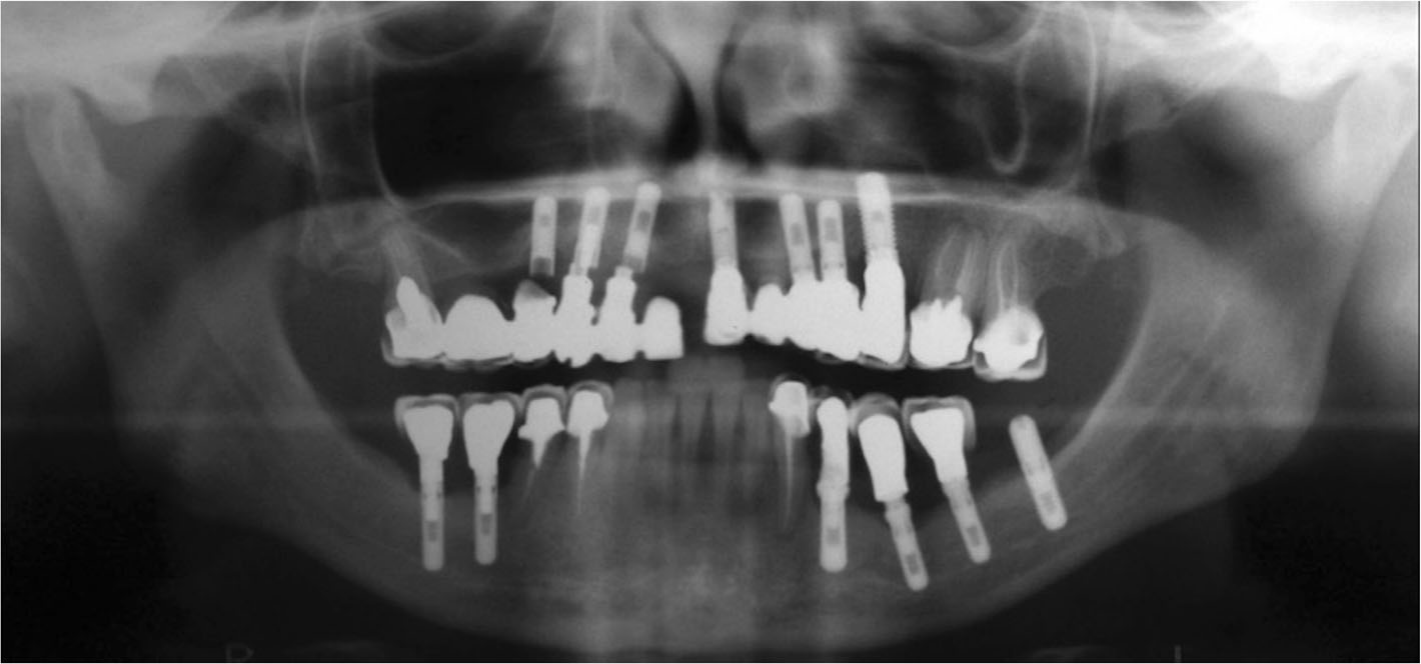
Radiograph of the fractured implants united to a tooth after 45 months in function

Three solid, commercially pure titanium screw-shaped implants (Colosso®, Emfils, Itu, São Paulo, Brazil) with surface sandblasted with 70–100 μm Al_2_O_3_ particles and etched by nitric acid, one of 3.3 mm in diameter and 11.5 mm long, and the other two of 3.3 mm in diameter and 10 mm long with internal hexagon, were retrieved from the 12, 13, and 14 teeth region of a 55-year-old woman’s maxilla. A six-element fixed restoration had been constructed connecting the three implants with a tooth on the second molar region (Fig. 1).

During a period of 3 years, the patient was recalled once a year, and no signs or symptoms of osseointegration loss were reported by her dentist. Forty-five months after implant placement, the abutment screws and the internal hexagon walls were fractured. There was no possibility to restore the implant/abutment connections.

Therefore, the implants and the surrounding bone were removed by using a 4-mm internal diameter trephine bur. The implant specimens were rinsed and immediately fixed in 4% neutral formalin for 48 h. Three new conic implants 5.0 mm wide and 10.00 mm long were placed immediately, and a sinus-lifting surgery was planned to put a fourth implant on the first molar region to avoid an implant/tooth union.

### Histologic processing

Undecalcified sections were prepared using the technique previously described by Donath and Breuner [12] (i.e.*,* the block was dehydrated by an ascending series of ethanol [60 to 100%], and it was embedded in glycolmethacrylate [Technovit 7200; Heraeus Kulzer GmbH, Wehrheim, Germany]). Subsequently, a ground section (40–50 μm) was obtained and stained with 1% toluidine blue.

### Qualitative and quantitative analysis

Through the histomorphometric analysis of the implants, the percentage of bone-to-implant contact (BIC%) and bone area formed between threads (BABT%) were obtained by a computerized method for histomorphometric analysis (UTHSCSA ImageTool® versão 3.0, Health Science Center, Texas University, EUA).

### Circularly polarized light microscopy

Birefringence was measured as an indicator of transverse collagen orientation using polarized light. Collagen fibers were viewed by placing the thin sections of bone under an Axioskop 40 microscope with circularly polarized light (Zeiss Oberkochen, Germany). Collagen fibers aligned in a perfect transverse way to the direction of the light propagation (perpendicular to the plane of the section) appeared “yellow-orange” because of a change in the refraction of exiting light, whereas the collagen fibers aligned along the axis of light propagation (parallel to the plane of the section) appeared “white-blue” because no refraction was present. The measurements of longitudinal or transverse collagen fiber areas to the quantitative analysis were made using the UTHSCSA ImageTool® versão 3.0 (Health Science Center, Texas University, EUA). To ensure accuracy, we calibrated the software for each experimental image.

## Results

**Fig. 2:**
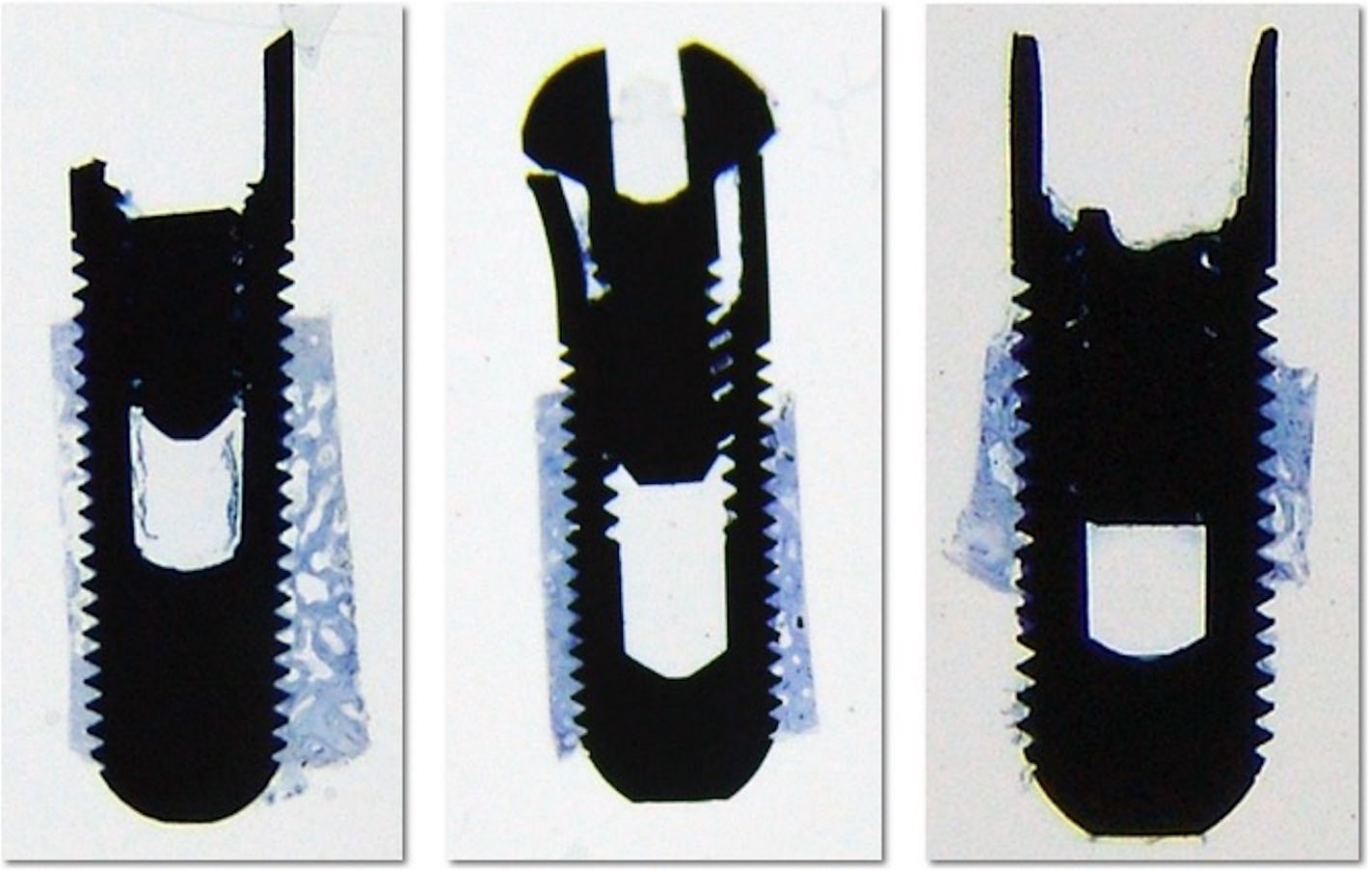
General overview of sandblasted and acid-etched implant histologic section. Note the presence of abutment screws and the internal hexagon walls which are fractured, and high bone density within threads of implant (toluidine blue, original magnification × 1.5)

The implants were completely surrounded by the new-formed bone that could not be differentiated from the original alveolus (Fig. 2).

**Fig. 3:**
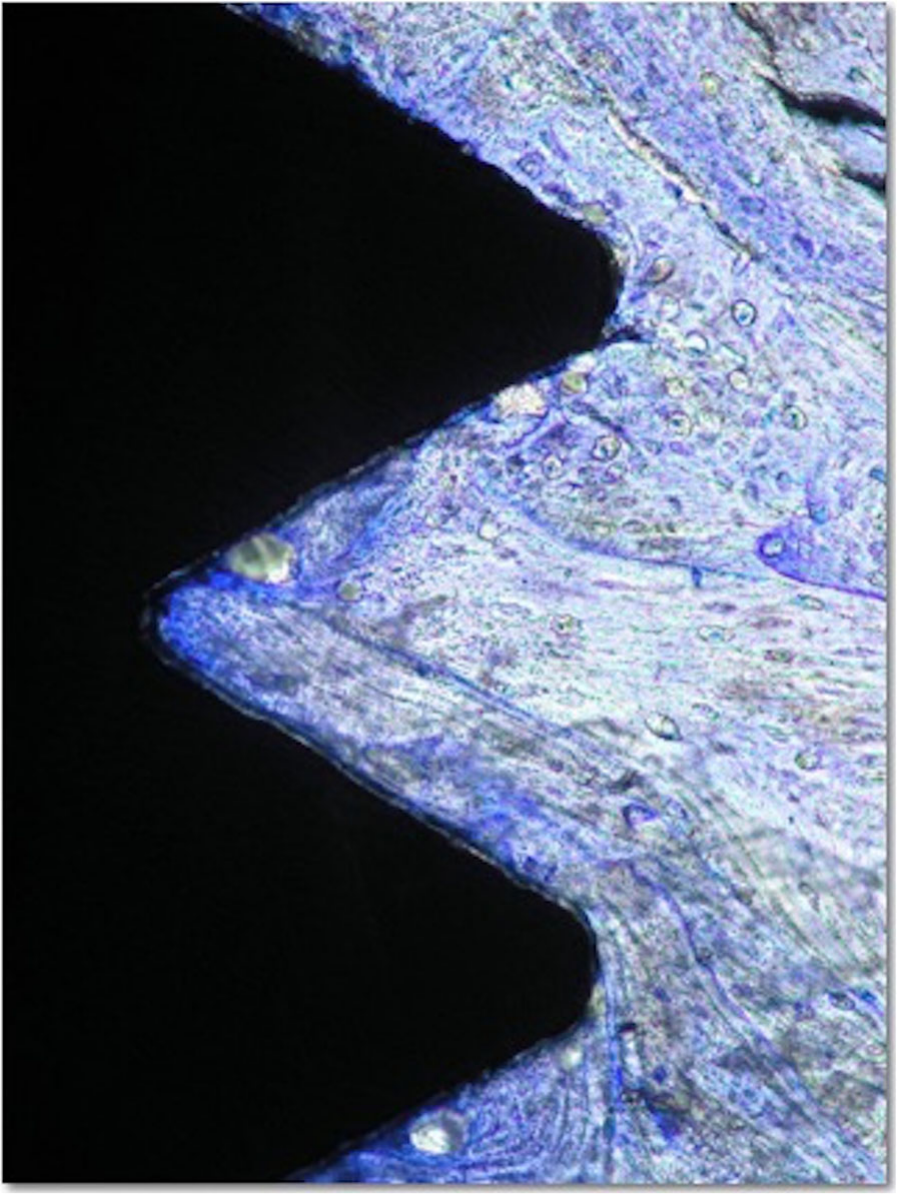
Histologic appearance of sandblasted and acid-etched implant. Direct bone contact with well-organized bone and lamellar apposition is observed

**Fig. 4:**
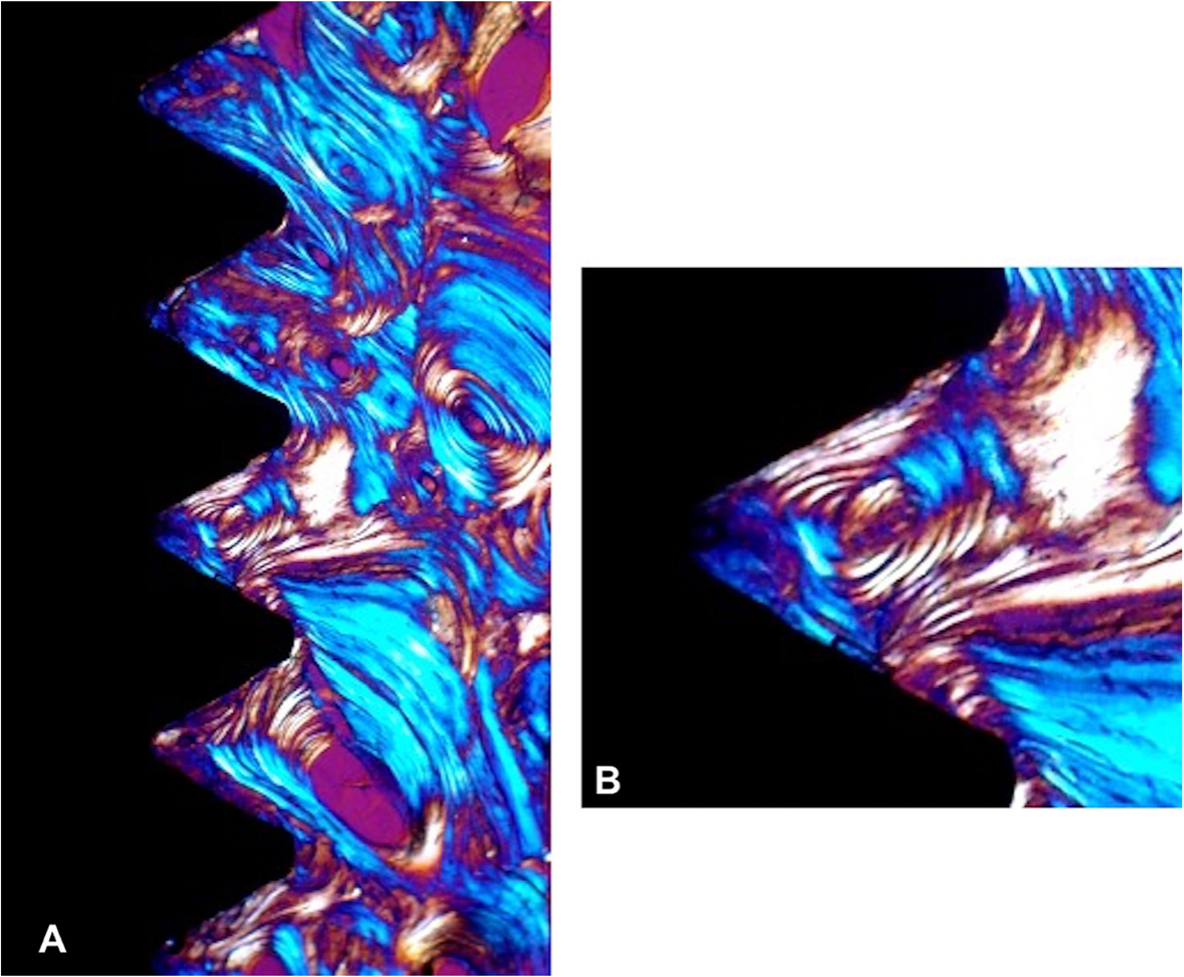
Under circularly polarized light microscopy (**a**) (original magnification × 50), the transverse collagen fibers appear in yellow-orange, while the longitudinal ones appear in white-blue. The transverse collagen fibers are mainly associated to the lower flank of the threads. **b** Original magnification × 200. Bone appears mainly constituted by transverse collagen fibers. The presence of an osteon shows bone remodeling activity

The bone surrounding the implant had a lamellar appearance, but the apposition of lamellae seemed to be not uniform, and some bone lacunae could be observed (Fig. 3). Osteons and osteocytes could be seen in direct contact with the porous surface of the implant. There were few gaps present in the interface, and the histomorphometric analysis revealed a mean bone-to-implant contact of 80.3% ± 4.1% (mean ± standard deviation) and a mean bone area of 77.3% ± 9.6% formed within the limits of the implant threads Orientation of the collagen fibers in the peri-implant bone was 63.14%, 40.91%, and 40.29% for transverse collagen fibers and 36.86%, 59.09%, and 59.71% for the longitudinal collagen fibers, respectively, for implants 12, 13, and 14 (Fig. 4).

**Fig. 5:**
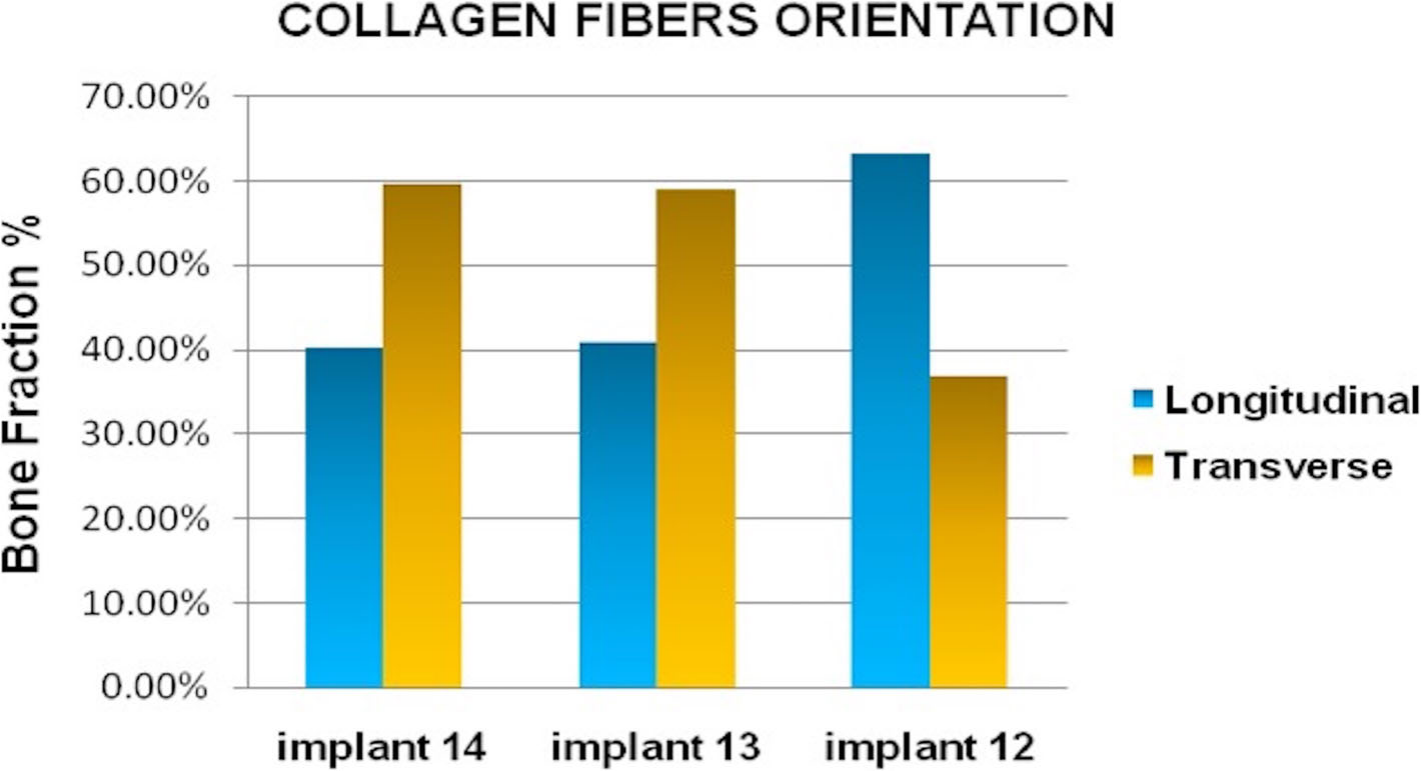
Percentage of the collagen fiber orientation of each implant

The comparison of the proportions between transverse and longitudinal collagen fibers revealed a high percentage of transverse fibers in contact with the implants positioned on the posterior region (Fig. 5). The histological observations on bone microstructure revealed an intense bone remodeling activity near the dental implant surface.

## Discussion

Histomorphometric analysis of human retrieved implants is the method available to analyze the bone-to-implant interface behavior over time [13]. The reproduction of a human’s mouth environment in animals is tremendously difficult. Therefore, this study can contribute to the knowledge of human bone response to a dental implant under loading conditions.

Many efforts have been made by researchers and manufacturers to produce implant surfaces attractive to living cells and, consequently, to improve the quantity and quality of osseointegration. It has been reported that micro-rough topography observed in a porous implant could favorably affect angiogenesis, as well as cellular migratory events, activity, and function [14], resulting in a faster and higher bone-implant contact and mechanical interlocking [6, 7].

In this study, three commercially pure, titanium screw-type implants were used. They formerly received a sandblasting treatment with aluminum oxide to promote macroporosities, and they were acid-etched to achieve microporosities. Rates of 80.3% of bone-to-implant contact and 77.3% of bone area within the limits of the implant threads were found. These findings are similar to the result reported by Hayakawa et al. [15] (76.60%) when a sandblasted and acid-etched implant was placed into the palatal bone as anchorage for orthodontic treatment.

Other investigators reported similar results with different surfaces and follow-up. Piattelli et al. [5] found 60 to 70% of bone-to-implant contact to titanium plasma spray implant. Brunel et al [16] reported 74% with hydroxyapatite coating in maxilla after 14 months of follow-up, and Degidi et al. [17] found 60% after 9 months of follow-up in porous anodized implant submitted to immediate loading.

Based on the trustworthiness of the macro- and microimplant systems, the implant-tooth splinting has been considered as an alternative in some clinical situations. Although some studies show satisfactory success in short and near future [18, 19], the previsibility of the implant/tooth system is still unclear.

The amount of tooth movement with healthy periodontal ligaments against that of an osseointegrated dental implant can be 5–20 times greater [20]. This disparity causes the implant side to receive a higher bending moment as a result of the bridge function as a cantilever construction and is only supported by the implant when the occlusal load acts on the tooth [21]. A series of potential problems such as tooth intrusion, osseointegration loss, screw loosening, and implant or prosthesis fracture can arise, with resulting complicated physiological and engineering aspects [18, 19, 22].

In the present case, three implants with 3.3 mm in diameter and with internal hexagon abutment connection were positioned at the 12, 13, and 14 teeth region and united to the second molar with reduced periodontal support, having the first molar suspended between them. As described by the literature, the occlusal load over the first and second molars resulted in a cantilever force that concentrated on the implant neck [23]. The association of overload, inadequate implant diameter to the case, and internal hexagon connection resulted in the abutment screw and the internal hexagon wall fracture.

The studies already showed the relation between the load and the collagen fiber orientation in bone near threaded dental [24, 25]. The spatial orientation of collagen fibers has a direct bearing on its mechanical properties [24]. Based on a number of studies, several authors also correlate strongly the collagen fiber orientation to the loading regimen [26, 27].

In 1958, Evans [28] described the relation between the bone stiffness and predominant direction of the collagen fibers in the bone matrix. When collagen fibers ran parallel to the loading vector, the bone was more resistant. McElhaney [29] found that the ultimate compressive strength and modulus of elasticity of the cortical bone increased with increasing strain rate. The energy absorption capacity had a maximum at an intermediate strain rate. It was suggested that low strain rate shear failures result from a distortion of the lamellar substructure and fracture along several weaker planes. High strain rate failures appeared to follow the cement lines, constituting the boundaries of the haversian and lamellar systems.

Traini et al. observed that the load can influence the collagen fiber orientation in bone near threaded dental implants in immediately loaded implants [24, 30]. They found that loading has a relevant influence in the distribution of the collagen fibers in the peri-implant bone. Transverse collagen fibers, related to compressive loads, were found in a higher and statistically significant quantity in loaded than in unloaded implants or in the alveolar bone. The bone tissue responded to an overloading (until the threshold of the implant fracture was reached) by modeling and remodeling its microstructure. The predominance of transverse collagen fiber orientation should be related to a high compression state [24, 30].

Regarding BIC and high load occlusion, Chang et al. [31] performed a systematic literature review and concluded that the greatest peri-implant bone remodeling activity is found around implants subjected to high loading forces, when the applied force exceeds the biological adaptable limit. The authors reported that there was a limitation in the research due to the absence of experimental studies in humans. But the authors suggest that a possible correlation between occlusal overload and implant failures is related to the degree tolerance of the alveolar bone which varies according to the individual, the location, and other anatomic and physiological parameters.

## Conclusion

Within the limitation of this study, the possible cause of the implant fracture could be the association of overload, inadequate implant diameter, and fragile internal hexagon connection.

## Data Availability

Not applicable

## Abbreviations

BABT: Bone area formed between threads
BIC: Bone-to-implant contact
